# Rules of engagement promote polarity in RNA trafficking

**DOI:** 10.1186/1471-2202-7-S1-S3

**Published:** 2006-10-30

**Authors:** John H Carson, Nicholas Blondin, George Korza

**Affiliations:** 1 Department of Molecular Microbial and Structural Biology, Center for Cell Analysis, and Modelling, University of Connecticut Health Center, Farmington, CT 06030, USA

## Abstract

Many cell biological pathways exhibit overall polarity (net movement of molecules in one direction) even though individual molecular interactions in the pathway are freely reversible. The A2 RNA trafficking pathway exhibits polarity in moving specific RNA molecules from the nucleus to localization sites in the myelin compartment of oligodendrocytes or dendritic spines in neurons. The A2 pathway is mediated by a ubiquitously expressed *trans*-acting trafficking factor (hnRNP A2) that interacts with a specific 11 nucleotide *cis*-acting trafficking sequence termed the A2 response element (A2RE) found in several localized RNAs. Five different molecular partners for hnRNP A2 have been identified in the A2 pathway: hnRNP A2 itself, transportin, A2RE RNA, TOG (tumor overexpressed gene) and hnRNP E1, each playing a key role in one particular step of the A2 pathway. Sequential interactions of hnRNP A2 with different molecular partners at each step mediate directed movement of trafficking intermediates along the pathway.

Specific "rules of engagement" (*both and*, *either or*, *only if*) govern sequential interactions of hnRNP A2 with each of its molecular partners. Rules of engagement are defined experimentally using three component binding assays to measure differential binding of hnRNP A2 to one partner in the presence of each of the other partners in the pathway. Here we describe rules of engagement for hnRNP A2 binding to each of its molecular partners and discuss how these rules of engagement promote polarity in the A2 RNA trafficking pathway.

For molecules with multiple binding partners, specific rules of engagement govern different molecular interactions. Rules of engagement are ultimately determined by structural relationships between binding sites on individual molecules. In the A2 RNA trafficking pathway rules of engagement governing interactions of hnRNP A2 with different binding partners provide the basis for polarity of movement of intermediates along the pathway.

## Background

Cell biological processes are mediated by pathways of interacting macromolecules. Comprehensive understanding of a particular cell biological pathway requires: inventory of all molecules involved in the pathway (including concentrations and diffusion coefficients); structures of all macromolecules in the pathway; interactions among molecules in the pathway (including kinetic constants); effects of perturbations of individual components of the pathway; and computational modeling of pathway behaviour [1]. Many of these requirements can be met using large scale systematic approaches. Large scale proteomics initiatives provide comprehensive inventories of molecules involved in different cell biological pathways. Large scale structure determination initiatives (X-ray crystallography and NMR) promise to provide detailed structural information on many individual pathway components. Large scale interaction screens (yeast two hybrid, GST pull down) can identify all interacting partners for each component of a pathway. Large scale functional genomics approaches using RNAi inhibition, antibody inhibition or mutagenesis can be used to knock out individual components in a pathway. Large scale modelling of systems of molecular interactions and complicated pathways can be accomplished using computer programs such as Virtual Cell  if quantitative system parameters are provided. These systematic approaches can provide detailed information about individual components and interactions in the pathway.

Many cell biological pathways contain key components that interact with multiple different molecular partners. In the overall human protein-protein interaction network some "hub" proteins have up to 30 different interacting partners [2]. Such proteins do not generally interact with all possible molecular partners simultaneously. Instead, binding interactions are regulated to ensure sequential binding and orderly progression of intermediates along the pathway(s). Possible regulatory mechanisms governing binding interactions include: sequestration of specific binding partners to particular subcellular compartments preventing interactions involving those binding partners in other compartments, post-translational modifications to specific binding partners altering their binding properties, and "rules of engagement" governing interactions of hub proteins with different binding partners. Here we discuss how rules of engagement regulate cell biological pathways.

Three general rules of engagement can be defined: *both and*, *either or*, and *only if *(equivalent to Boolean operators OR, XOR and AND, respectively). The *both and *rule states that *A *can interact with *both B and C*, simultaneously (as well as individually). The *either or *rule states that *A *can interact with *either B or C*, but not with both simultaneously. The *only if *rule states that *A *can interact with *B only if C *also interacts (this implies a quantitative rather than absolute difference in binding since A can bind to both B and C, individually). Rules of engagement have important implications for computational modelling of cell biological pathways. If *A *can bind *either B or C*, the model must include the following reactions: *A *+ *B **AB *and *A *+ *C **AC*. However, if *A *can bind to *both B and C*, simultaneously, the following additional reactions must be included in the model: *AB *+ *C **ABC *and *AC *+ *B **ABC*. If *A *binds to *B only if C *also binds, separate kinetic constants must be specified for *A *+ *B **AB *and *AC *+ *B **ABC*. Thus, understanding rules of engagement is essential for computational modelling of cell biological pathways.

Rules of engagement have specific implications regarding binding sites for different partners on the target molecule. If *A *binds *both B and C *simultaneously this implies that *A *contains binding sites for both *B *and *C *that are independent of each other. If *A *binds *either B or C*, but not both, this implies either that *A *contains binding sites for *B *and *C *that overlap so that both molecules compete for the same site and cannot bind simultaneously (competitive inhibition), or that binding of *B *induces a conformational change in *A *that prevents binding to *C *(non-competitive inhibition). If *A *binds *B only if C *also binds, this implies that binding of *B *induces a conformational change in *A *that facilitates binding of *C*. Elucidating the structural basis for specific rule of engagement will ultimately require detailed analysis of the binding surfaces on each molecule. However, in the absence of such detailed structural data, experimentally determined rules of engagement can provide important insight into relationships among binding sites on interacting molecules.

In principle, rules of engagement can be determined experimentally using any three component system where binding between two components can be quantified in the presence or absence of the third component. If binding of *A *to *B *is not affected by binding to *C*, this indicates that *A *can interact with *both B and C*, simultaneously. If binding of *A *to *B *is inhibited by binding to *C*, this indicates that *A *can interact with *either B or C *but not with both simultaneously. If binding of *A *to *B *stimulates binding to *C*, this indicates that *A *can interact with *B only if C *also binds. Measuring binding between each pairwise combination of interacting partners in the presence of each of the other binding partners generates a table defining rules of engagement for interactions among different binding partners.

In the work described here rules of engagement are determined using fluorescence correlation spectroscopy (FCS) to measure binding of differentially labelled fluorescent recombinant proteins in three component binding assays. In FCS, fluorescent fluctuations that occur as fluorescent molecules traverse a small (< 1 fl) observation volume are analyzed by correlation techniques to determine concentrations and diffusion times [3]. Autocorrelation analysis of individual channels separately provides a measure of the total concentration of each fluorescent molecule. Cross correlation analysis of the two channels provides a measure of the concentration of molecules that interact with each other. To determine rules of engagement by FCS the target protein and one specific partner are differentially labelled with fluorophores. Binding is measured by cross correlation FCS in the presence and absence of each of the other unlabeled partners. If the unlabeled binding partner does not affect the amount of cross correlation, this indicates that the target protein can bind *both *the labelled partner *and *the unlabeled partner simultaneously. If the unlabeled partner reduces the amount of cross correlation, this indicates that the target protein binds *either *the labelled partner *or *the unlabeled partner. If the unlabeled protein increases the amount of cross correlation, this indicates that the target protein binds the labelled partner *only if *the unlabeled partner also binds. In this manuscript we will summarize rules of engagement for molecules in the A2 RNA trafficking pathway [4]. Detailed description of the FCS experiments used to determine rules of engagement for specific molecular interactions will be published separately.

**Figure 1:**
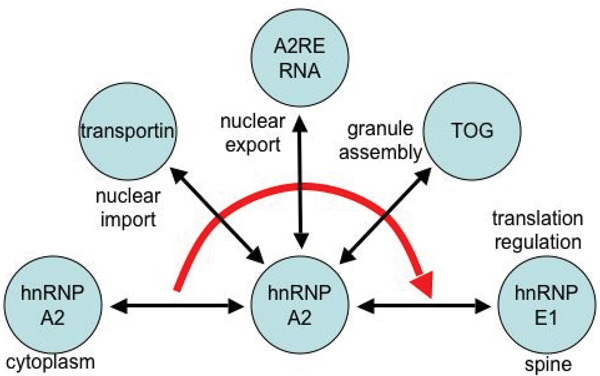
**Molecular interactions of hnRNP A2 in the A2 RNA trafficking pathway**. Five different molecular partners of hnRNP A2 have been identified in the A2 RNA trafficking pathway – hnRNP A2 itself, transportin 1, A2RE RNA, TOG and hnRNP E1. Each step of the A2 RNA trafficking pathway is mediated by interaction of hnRNP A2 with a particular partner. Although interactions between hnRNP A2 and each partner are reversible, the overall pathway exhibits polarity.

Rules of engagement promote polarity in cell biological pathways by facilitating interactions that mediate movement of intermediates in one direction and inhibiting interactions that mediate movement in the reverse direction. Here we will discuss how rules of engagement governing molecular interactions of hnRNP A2 with different molecular partners promote polarity in the A2 RNA trafficking pathway [4]. The A2 RNA trafficking pathway targets specific RNAs to the myelin compartment in oligodendrocytes or to dendritic spines in neurons, maximizing expression of the encoded proteins in the subcellular compartment where the RNA is localized and minimizing ectopic expression elsewhere in the cell. The key *trans*-acting factor in the A2 pathway is hnRNP A2, which binds specifically to an 11 nucleotide *cis*-acting sequence (GCCAAGGAGCC), termed the A2RE (hnRNP A2 response element) found in several localized RNAs. HnRNP A2 is exported from the nucleus in association with A2RE RNA as part of an "RNP code" consisting of an ensemble of specific RNA binding proteins that determines subsequent processing, trafficking, translation and degradation of the RNA. As the RNA moves from one compartment to the next, certain proteins dissociate and others bind. However, hnRNP A2 remains associated with A2RE RNA throughout the A2 pathway and plays key roles at each step in the pathway by binding to a series of different molecular partners. The overall pathway can be decomposed into six steps: nuclear import of hnRNP A2; binding of hnRNP A2 to A2RE RNA in the nucleus, nuclear export of hnRNP A2 bound to A2RE RNA; assembly of hnRNP A2::A2RE RNA complexes into large supramolecular trafficking intermediates termed RNA granules; bi-directional transport of hnRNP A2::A2RE RNA granules along microtubules, during which translation is inhibited; and translation of RNA granules at localization sites (myelin compartment in oligodendrocytes, dendritic spines in neurons). As shown in figure 1, five different molecular partners for hnRNP A2 have been identified in the A2 pathway: hnRNP A2 itself, transportin 1, A2RE RNA, TOG protein and hnRNP E1. Transition of trafficking intermediates from one step to the next involves sequential binding of hnRNP A2 to different molecular partners at each step. Here we show that polarity in the A2 pathway is determined by rules of engagement governing specific interactions of hnRNP A2 with individual components of the A2 pathway.

## Results and Discussion

### hnRNP A2 binding to hnRNP A2

**Figure 2:**
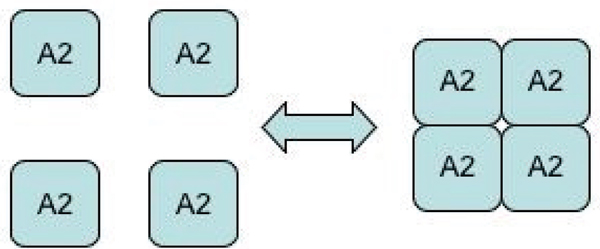
**hnRNP A2 forms oligomers**. Individual hnRNP A2 molecules form homotypic interactions with other hnRNP A2 molecules to generate oligomers.

HnRNP A2 is a 36 kD RNA binding protein that shuttles between nucleus and cytoplasm [14], interacting with different partners at each step in the A2 RNA trafficking pathway [4]. The protein contains two RNA recognition motif (RRM) domains, followed by a glycine rich domain (GRD) and an M9 nuclear localization domain [6,8]. Homotypic interaction of hnRNP A2 with itself, mediated by the GRD, has been demonstrated by yeast two hybrid analysis [5]. Fluorescence correlation spectroscopy (FCS) studies indicate that hnRNP A2 forms oligomers (predominantly tetramers) in solution (apparent K_d _~ 0.1 nM), which implies that each hnRNP A2 molecule contains at least two separate homotypic binding sites, one mediating association of monomers to form dimers and one mediating association of dimers to form higher order aggregates (figure 2). Thus, each hnRNP A2 molecule interacts with *both *hnRNP A2 to form dimers *and *a second hnRNP A2 to form tetramers, indicating that each hnRNP A2 molecule contains two separate homotypic binding sites. In most cells, the concentration of hnRNP A2, determined by quantitative western blotting, is 1–6 μM in cytoplasm, and ~20 fold higher in nucleus [8,18]. At these concentrations it is likely that most hnRNP A2 in the cell is present as oligomers, unless binding to other binding partners prevents oligomer formation.

To determine rules of engagement governing interactions of hnRNP A2 with itself and with each of the other partners, the oligomeric state of hnRNP A2 in the presence of each of its different binding partners was analyzed by FCS. The oligomeric state of hnRNP A2 was not affected by transportin, A2RE RNA or hnRNP E1, indicating that hnRNP A2 can interact with *both *itself *and *transportin, A2RE RNA or hnRNP E1. This means that homotypic binding sites in hnRNP A2 do not overlap with binding sites for transportin, A2RE RNA or hnRNP E1. However, addition of subfragments of TOG caused dissociation of hnRNP A2 oligomers to dimers, indicating that hnRNP A2 dimers bind to *either *other hnRNP A2 dimers *or *to subfragments of TOG, but not to both simultaneously. This means that the binding site for TOG on the hnRNP A2 molecule overlaps with one of the homotypic binding sites.

### hnRNP A2 binding to transportin 1

Transportin is a 97 kD karyopherin that mediates nuclear import of proteins such as hnRNP A2 that contain non-conventional nuclear localization signals [6]. The M9 nuclear localization signal in hnRNP A2, consisting of ~35 amino acids near the carboxy terminus of the protein, binds to a cluster of HEAT repeat motifs in the carboxy terminal half of transportin [12]. During translocation of transportin::hnRNP A2 from cytoplasm to nucleus transportin interacts with nucleoporins in the nuclear pore complex (NPC). In the nucleus, transportin dissociates from hnRNP A2 and binds to Ran GTP. Transportin::RanGTP is then exported from nucleus to cytoplasm through the NPC [16]. Thus, during nuclear import transportin interacts with *both *hnRNP A2 *and *nucleoporins, in the nucleus transportin interacts with *either *hnRNP A2 *or *RanGTP, and during nuclear export transportin interacts with *both *RanGTP *and *nucleoporins. The high nuclear RanGTP concentration favors formation of transportin::RanGTP in the nucleus, which makes nuclear import of hnRNP A2 by transportin essentially irreversible. FCS experiments indicate that hnRNP A2 can bind *both *other hnRNP A2 molecules *and *transportin, simultaneously. It is not known if hnRNP A2 is transported into the nucleus as a monomer or as an oligomer. Nor is it known if a single transportin molecule is sufficient to translocate an hnRNP A2 oligomer into the nucleus or whether separate transportin molecules bind to each hnRNP A2 molecule in the oligomer during nuclear import.

As discussed in the following section, hnRNP A2 binding to A2RE RNA inhibits hnRNP A2 binding to transportin, indicating that hnRNP A2 can bind *either *A2RE RNA *or *transportin, but not both simultaneously. Since the RNA binding sites in hnRNP A2 are located in the N terminal portion of the molecule [7] while the transportin binding site is located near the C terminus [12], the binding sites do not overlap in the primary sequence. Binding of A2RE RNA may cause a conformational change in the hnRNP A2 protein that interferes with binding of transportin. Subfragments of TOG also inhibit binding of hnRNP A2 to transportin (data not shown), indicating that hnRNP A2 can bind *either *transportin *or *TOG but not both simultaneously. Both transportin and TOG protein contain multiple clusters of HEAT repeats [9,12]. The hnRNP A2 binding site(s) in TOG has not been identified. However, since HEAT repeats in transportin bind to the M9 domain of hnRNP A2 it is possible that HEAT repeats in TOG also bind to the M9 domain of hnRNP A2. Thus, transportin and TOG may compete for the same binding site in hnRNP A2, resulting in binding of hnRNP A2 to *either *transportin *or *TOG but not both. hnRNP E1 does not affect binding of hnRNP A2 to transportin, indicating that hnRNP A2 can bind *both *transportin *and *hnRNP E1 simultaneously.

### hnRNP A2 binding to A2RE RNA

In the nucleus, hnRNP A2 dissociates from transportin and binds to A2RE RNA. Surface plasmon resonance (SPR) analysis indicates that hnRNP A2 contains two RNA binding sites – a specific site, which binds to the A2RE sequence (GCCAAGGAGCC) (apparent K_d _~ 50 nM) and a non specific RNA binding site (apparent K_d _~ 300 nM) [7]. Both sites are found in the N terminal portion of the hnRNP A2 protein in a region consisting of two consensus RRM domains. Because of the high concentration of hnRNP A2 in the nucleus, it is possible that each A2RE RNA molecule interacts with multiple hnRNP A2 molecules *both *specifically at A2RE sites *and *non-specifically, elsewhere on the RNA. Binding of A2RE RNA does not affect the oligomeric state of hnRNP A2, indicating that hnRNP A2 can bind *both *RNA *and *other hnRNP A2 molecules simultaneously. This means that the RNA binding sites in hnRNP A2 do not overlap with the oligomerization sites.

The effect of transportin on binding of hnRNP A2 to A2RE RNA has not been determined *in vitro*. However, in oligodendroyctes in culture, hnRNP A2-GFP expressed by itself is imported efficiently into the nucleus, presumably through binding to transportin, whereas in the presence of excess A2RE RNA, hnRNP A2-GFP is retained in the cytoplasm [8]. This suggests that binding of hnRNP A2 to A2RE RNA prevents binding to transportin, which means that hnRNP A2 can bind *either *A2RE RNA *or *transportin, but not both molecules simultaneously. As discussed above, since binding sites for transportin and A2RE RNA are physically separated within the hnRNP A2 molecule, this means that binding one partner (A2RE RNA) induces a conformational changes in hnRNP A2 that interferes with binding of the other partner (transportin). The effect of hnRNP E1 on binding of hnRNP A2 to A2RE RNA also has not been determined *in vitro*. However, hnRNP E1 is co-localized in granules with hnRNP A2 and A2RE RNA, and hnRNP E1 inhibits translation of A2RE RNA in an hnRNP A2-dependent manner [18], suggesting that hnRNP A2 can bind *both *A2RE RNA *and *hnRNP E1, simultaneously. This means that the RNA binding sites in hnRNP A2 do not overlap the hnRNP E1 binding site(s).

### hnRNP A2 binding to TOG protein

**Figure 3:**
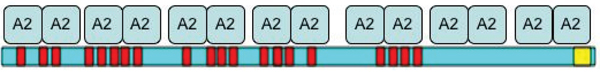
**TOG contains multiple hnRNP A2 binding sites**. The TOG protein is depicted in cyan with identified HEAT repeats in red and an NTF2 homology domain in yellow. hnRNP A2 dimers ar shown in light blue. Seven subfragments of TOG protein were cloned, each corresponding to a cluster of HEAT repeats or alpha helical domains. Each subfragment of TOG showed binding to hnRNP A2. Binding to TOG subfragments caused dissociation of hnRNP A2 oligomers to dimers.

Tumor overexpressed gene (TOG) protein is a long (215 kD) filamentous microtuble binding protein [13]. TOG orthologues in species from yeast to humans are implicated in regulation of microtubule stability during mitosis [15]. TOG was identified as a binding partner for hnRNP A2 in a yeast two hybrid screen [9]. In oligodendrocytes TOG protein is co-localized with hnRNP A2 in A2RE RNA granules [9]. Sequence comparisons have identified five clusters of HEAT repeats (helix turn helix motifs) in TOG protein [15] and secondary structure algorithms predict a high probability of alpha helical structure throughout the length of the TOG protein. The TOG sequence can be divided into seven subfragments corresponding to the five identified HEAT repeat clusters from the N terminal portion of the protein and two alpha helix rich regions from the C terminus of the protein. Each subfragment was cloned, expressed in bacteria and tested for binding to hnRNP A2 by FCS and in vitro cross-linking. The results indicate that each of the seven subfragments of TOG binds to hnRNP A2. This suggests that full length TOG protein represents a scaffold for binding a linear array of hnRNP A2 molecules, as illustrated in figure 3. This could provide the basis for assembly of multiple A2RE RNA molecules into granules in the A2 pathway.

FCS experiments indicate that binding of individual TOG subfragments causes dissociation of hnRNP A2 oligomers to dimers, indicating that each dimer of hnRNP A2 can bind to *either *another dimer of hnRNP A2 to form a tetramer, *or *to TOG protein. TOG and transportin appear to compete for the same binding sites in hnRNP A2, indicating that hnRNP A2 can bind *either *TOG *or *transportin. However, transportin does not cause dissociation of hnRNP A2 oligomers to dimers the way TOG does, suggesting that the binding sites for transportin and TOG are not completely congruent. Binding of hnRNP A2 to TOG is not affected by hnRNP E1, indicating that hnRNP A2 can bind *both *TOG *and *hnRNP E1, simultaneously. This means that the binding sites for TOG and hnRNP E1 do not overlap.

### hnRNP A2 binding to hnRNP E1

hnRNP E1 is a 40 kD RNA binding protein containing three KH domains [17]. Together with hnRNP K, hnRNP E1 binds specifically to a *cis*-acting poly pyrimidine translation regulation element called DICE, found in several different translationally regulated RNAs [10]. Binding of hnRNP E1 and/or hnRNP K to the DICE element in lipoxygenase RNA inhibits translation by interfering with recruitment of the 60S ribosomal subunit. A2RE RNAs are not known to contain DICE elements. However, hnRNP E1 binds to hnRNP A2 and is co-localized with hnRNP A2 in A2RE RNA granules in oligodendrocytes. Furthermore, hnRNP E1 inhibits translation of A2RE RNA *in vitro *and *in vivo*, in an hnRNP A2-dependent fashion, suggesting that hnRNP A2 bound to A2RE RNA recruits hnRNP E1, which inhibits translation of the RNA during transport [18].

Binding of hnRNP E1 does not affect the oligomeric state of hnRNP A2, indicating that hnRNP A2 can bind *both *itself *and *hnRNP E1, simultaneously. Since hnRNP E1 is co-localized with hnRNP A2 in A2RE granules during transport, it is likely that hnRNP A2 can bind *both *A2RE RNA *and *hnRNP E1 simultaneously. Binding of transportin or TOG to hnRNP A2 is not affected by hnRNP E1, indicating that hnRNP A2 can bind *both *hnRNP E1 *and *TOG or transportin. Thus, rules of engagement for hnRNP A2 binding to hnRNP E1 are *both and *with respect to each of the other hnRNP A2 binding partners, which means that hnRNP E1 binding sites on hnRNP A2 are independent of binding sites for each of its other binding partners. Analysis of binding between subfragments of hnRNP A2 and hnRNP E1 by FCCS indicates that both N and C terminal subfragments of both proteins contain separate binding sites for the other protein [18]. Since hnRNP A2 can bind to hnRNP E1 through multiple alternative binding surfaces it is likely that binding sites for each of the other hnRNP A2 binding partners will be independent of at least one of the hnRNP E1 binding surfaces, providing an explanation for the *both and *rule of engagement governing binding of hnRNP A2 and hnRNP E1 with respect to each of the other hnRNP A2 binding partners.

### Rules of engagement for hnRNP A2 interactions

Rules of engagement for hnRNP A2 molecular interactions are summarized in Table 1. The table contains *both and *interactions and *either or *interactions but does not contain *only if *interactions. Most of the interactions in the table have been analyzed using three component *in vitro *binding experiments with bacterially expressed recombinant proteins. It is possible that in living cells rules of engagement are altered by partitioning of specific binding partners to particular subcellular compartments, post-translational modifications of specific binding partners or by binding to other partners, not shown in the table. Nevertheless, rules of engagement determined *in vitro *provide important insight into the A2 RNA trafficking pathway. In the following sections we discuss molecular interactions of hnRNP A2 at each step in the A2 pathway with a view to understanding how rules of engagement determine polarity.

### Nuclear import of hnRNP A2

Nuclear import of hnRNP A2 is mediated by transportin [6]. Movement of transportin::hnRNP A2 through the NPC is facilitated by transient interactions of transportin with nucleoporins in the NPC [16]. Most nuclear transport models assume that transportin::hnRNP A2 can move in either direction through the NPC so that this step does not have intrinsic polarity. However, the nucleus contains alternative *either or *binding partners for both hnRNP A2 (A2RE RNA) and transportin (RanGTP) so that if transportin::hnRNP A2 dissociates in the nucleus hnRNP A2 will likely bind to A2RE RNA and transportin will likely bind to RanGTP, preventing their reassociation with each other. HnRNP A2 associated with A2RE RNA will be exported from the nucleus via the mRNA export pathway [11] while transportin::RanGTP can move through the NPC by interaction with nucleoporins. In the cytoplasm, the GTPase activity of Ran is activated by RanGAP, converting RanGTP to RanGDP, which dissociates from transportin, freeing it for another round of nuclear import. Thus, although movement of transportin::hnRNP A2 through the NPC may lack polarity, overall nuclear import of hnRNP A2 by transportin has polarity because *either or *rules of engagement govern interactions of both transportin and hnRNP A2 with other binding partners that are sequestered in the nucleus. Overall polarity of nuclear import generates a nuclear/cytoplasmic partition coefficient of ~20:1 for hnRNP A2 [8].

### Nuclear export of hnRNP A2

The high concentration of hnRNP A2 in the nucleus means that most nuclear hnRNP A2 is probably oligomeric. Both oligodendrocytes and neurons (and probably most other cells) express multiple different A2RE RNAs that are transported by the A2 pathway. Since hnRNP A2 can *both *oligomerize *and *bind A2RE RNA simultaneously, it is likely that in the nucleus each A2RE RNA molecule associates with multiple hnRNP A2 oligomers (and other RNP molecules) through specific and non-specific binding interactions. Since these interactions are governed by *both and *rules of engagement they do not contribute to polarity in the A2 pathway.

The detailed molecular mechanism for nuclear export of hnRNP A2 oligomers associated with A2RE RNAs has not been determined but is likely to follow the mRNA export pathway [11]. When hnRNP A2 oligomers associated with A2RE RNA reach the cytoplasm they can interact with TOG protein, which is excluded from the nucleus during interphase. Interaction with TOG has two consequences for hnRNP A2. First, hnRNP A2 oligomers are converted to dimers because hnRNP A2 dimers can bind *either *to TOG *or *to other A2 dimers, but not both. Second, because TOG is a long filamentous molecule [13] containing a linear array of hnRNP A2 binding sites, each of which can bind an hnRNP A2 dimer with associated A2RE RNA, multiple hnRNP A2::A2RE RNA complexes will be organized in a linear array by binding to adjacent binding sites on the TOG protein. This may nucleate granule assembly and effectively block reuptake of TOG-associated hnRNP A2 into the nucleus because the TOG::hnRNP A2::A2RE RNA complex may be too large to pass through the NPC. Furthermore, since hnRNP A2 can bind *either *TOG *or *transportin, but not both, binding to TOG precludes binding to transportin, which is necessary for nuclear import. Thus, *either or *rules of engagement governing hnRNP A2 binding to TOG and transportin, ensure polarity in nuclear export of hnRNP A2 associated with A2RE RNA.

### Assembly of hnRNP A2::A2RE RNA into granules

The process of granule assembly requires controlled aggregation of RNA and protein components into large supramolecular assemblies. Each granule contains multiple (up to 30) RNA molecules, cognate RNA binding proteins (hnRNP A2, hnRNP E1), TOG protein, molecular motors (conventional kinesin and dynein), and components of the translational machinery. RNA granules assemble rapidly (minutes) and appear to be relatively stable even though individual binding interactions among granule components are freely reversible. The explanation for the stability of RNA granules may lie in the multiplicity of binding interactions of hnRNP A2 and rules of engagement governing those interactions. Each hnRNP A2 molecule has multiple potential interactions including: at least two different homotypic interactions, specific and non-specific binding sites for A2RE RNA, four possible binding interactions with hnRNP E1 and seven different binding interactions with TOG protein. If granule assembly is considered as molecular aggregation the rate of assembly is directly proportional to the number of unoccupied binding sites in the aggregate while the rate of disassembly is inversely proportional to the number of occupied binding sites per subunit. For example, a molecule containing two homotypic binding sites can form aggregate through head-to-tail interactions. However, the total number of available binding sites for the aggregate (which determines the rate of aggregation) will never increase above two, whereas the number of possible sites for dissociation (which determines the rate of disaggregation) will increase in proportion to the number of subunits in the aggregate. In other words, subunits with only two binding sites cannot form very large aggregates. However, if the number of binding sites per subunit is larger, the total number of available binding sites per aggregate will increase with aggregate size. If individual subunits interact with more than two other subunits in the aggregate the overall dissociation rate will decrease in proportion to the number of interactions formed. Thus, increasing the number of binding sites per subunit increases the aggregation rate and decreases the disaggregation rate. By this reasoning, the multiplicity of binding sites on each hnRNP A2 molecule and the variety of potential interactions will tend to increase both the rate of granule assembly and the size and the stability of granules formed. The exact stoichiometric molecular composition and size of each granule may vary somewhat depending upon which particular molecular interactions of hnRNP A2 occur during the process of granule assembly, introducing an element of stochasticity in granule assembly.

### Transport of granules on microtubules

Each granule contains both plus end (conventional kinesin) and minus end (cytoplasmic dynein) molecular motors which drive bi-directional motion along microtubules. NonA2RE RNA granules are retained in the perikaryon where the RNA is translated, whereas A2RE RNA granules are transported to localization sites in the cell periphery (the myelin compartment in oligodendrocytes, dendritic spines in neurons) where the RNA is translated. It is not known whether differential distribution of A2RE and nonA2RE RNAs is predominantly due to differential transport on microtubules or to differential capture at localization sites, or both. In the differential transport model, the RNA cargo of the granule somehow affects motor activities associated with the granule to generate overall movement biased towards either the plus end (for A2RE RNA granules) or minus end (for nonA2RE RNA granules) of the microtubule. The basis for differential regulation of motor activities is not known. In the differential capture model, granules move bidirectionally in the dendrites. A2RE RNA granules are captured at specific localization sites in the myelin compartment of oligodendrocytes or at dendrtic spines in neurons, while nonA2RE RNA granules are either captured at specific sites in the perikaryon or remain in the perikaryon by default. The mechanism for differential capture at specific sites is not known. The basis for polarity in differential localization of A2RE RNA versus nonA2RE RNA granules, whether mediated by differential transport and differential capture, is not known.

### Translation regulation

Translation is suppressed during RNA granule transport and activated upon granule localization. Translation of A2RE RNA is suppressed by hnRNP E1, which binds to hnRNP A2. During red cell differentiation translation of lipoxygenase RNA is suppressed by hnRNP E1 and hnRNP K, which block recruitment of the 60S ribosomal subunit. Translation of lipoxygenase RNA is activated when hnRNP E1 and K dissociate from the RNA allowing recruitment of 60S ribosomal subunit and polysome formation. The mechanism of translation suppression during transport of A2RE RNA may be similar, since hnRNP A2 binds to *both *A2RE RNA *and *hnRNP E1. Recruitment of hnRNP E1 to A2RE RNA may be mediated through binding of hnRNP E1 to hnRNP A2 associated with A2RE RNA, which could occur in the nucleus, before export of the RNA to the cytoplasm, ensuring that A2RE RNA is not translated in the perikaryon prior to localization. When A2RE RNA granules are localized in the periphery (in the myelin compartment of oligodendroctyes or in dendritic spines of neurons) hnRNP E1 dissociates by an unknown mechanism. In the case of lipoxygenase RNA in red cells, phosphorylation of hnRNP K causes dissociation. A similar mechanism may mediate hnRNP E1 dissociation from A2RE RNA granules. If hnRNP E1 (or hnRNP A2) is phosphorylated by a kinase (or other post-translational modification) localized to the myelin compartment of oligodendrocytes or dendritic spines of neurons, the rules of engagement governing binding of hnRNP A2 to A2RE RNA and hnRNP E1 may be altered resulting in dissociation of hnRNP E1 from hnRNP A2, allowing recruitment of 60S ribosomal subunit and formation of polysomes on A2RE RNA. In this way, translation activation may represent a polarity step in the A2 pathway mediated by alteration in the rules of engagement governing interaction of hnRNP A2 with A2RE RNA and hnRNP E1.

## Conclusion

**Figure 4:**
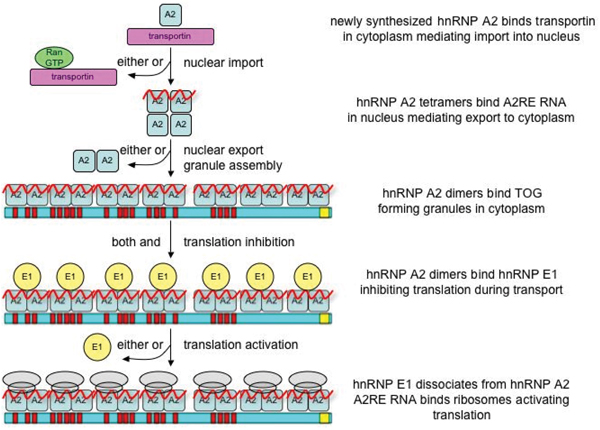
**Sequential molecular interactions in the A2 pathway**. Molecular interactions associated with sequential steps in the A2 pathway are illustrated. In the first step, newly synthesized hnRNP A2 binds to transportin in the cytoplasm and is imported into the nucleus. In the nucleus, transportin binds *either *RanGTP *or *hnRNP A2 and hnRNP A2 binds *either *A2RE RNA *or *tansportin. In the next step, hnRNP A2 oligomers bind to A2RE RNA in the nucleus and are exported to the cytoplasm. In the cytoplasm, hnRNP A2 dimers bind *either *to other hnRNP A2 dimers *or *to TOG protein, which mediates granule assembly. In the next step, hnRNP A2 binds *both *TOG protein *and *hnRNP E1, which inhibits translation during transport. In the last step, granules bind *either *hnRNP E1 *or *ribosomal subunits, to initiate translation. Steps involving *either or *interactions are essentially irreversible and promote polarity to the pathway. Steps involving *both and *interactions are reversible.

The A2 RNA trafficking pathway is mediated by sequential interactions of hnRNP A2 with five different molecular partners. Although each interaction in the pathway is reversible, the overall pathway has polarity because certain steps in the pathway are rendered irreversible by rules of engagement governing interaction of hnRNP A2 with specific individual molecular partners in the presence of other partners. Rules of engagement for hnRNP A2 have been determined experimentally in three component binding assays. Several interactions of hnRNP A2 are governed by *both and *rules, which do not affect polarity, while, other interactions governed by *either or *rules generate polarity by making specific steps in the pathway essentially irreversible. Sequential molecular interaction in the A2 pathway are outlined in Figure 4. Polarity in other cell biological pathways containing hub proteins with multiple different molecular partners may also reflect rules of engagement governing different interactions. In such cases, determining rules of engagement for molecular interactions of hub proteins may provide important insight into the basis for polarity.

## Authors' contributions

The work described in this manuscript was performed by George Korza and Nicholas Blondin in the laboratory of Dr. John Carson. The manuscript was drafted by Dr. Carson.

## Figures and Tables

**Table 1:** 

	A2	TRN	A2RE	cTOG	E1
A2::A2	*both and*	*both and*	*both and*	*either or*	*both and*
A2::TRN	*both and*	*both and*	*either or*	*either or*	*both and*
A2::A2RE	*both and*	*either or*	*both and*	*either or*	*both and*
A2::TOG	*either or*	*either or*	*either or*	*either or*	*both and*
A2::E1	*both and*	*both and*	*both and*	*both and*	*both and*
